# Recent advances in polysaccharides derived from the *Dendrobium nobile* Lindl.: preparation strategies, structural characteristics, biological activity, and structure-activity relationships

**DOI:** 10.3389/fphar.2025.1631637

**Published:** 2025-07-24

**Authors:** Xiao Zhou, Haizheng Bi, Zhaojiong Zhang, Xingyu Wang, Mengru Zhang, Meng Wang

**Affiliations:** Key Laboratory of Basic and Application Research of Beiyao Ministry of Education, Heilongjiang University of Chinese Medicine, Harbin, China

**Keywords:** *Dendrobium nobile* Lindl., polysaccharides, extraction, purification, structural characteristics, biological activity

## Abstract

*Dendrobium nobile* Lindl. (*D. nobile*) has significant medicinal value. *D. nobile* is used in traditional Chinese medicine and is widely popular as a functional food and health supplement due to its nourishing properties and high safety. Among its key bioactive constituents, polysaccharides exhibit promising applications across medicine, personal care, food, and agriculture, owing to their anti-photoaging, improvement of complications of diabetes mellitus, ovarian protective, gastric protective, neuroprotective, anti-inflammatory and anti-viral effects. Despite these multifaceted benefits, research on *D. nobile* polysaccharides remains limited relative to more extensively studied components such as alkaloids and flavonoids. This review systematically summarizes current advances in extraction techniques, structural features, bioactivities, and structure–activity relationships of *D. nobile* polysaccharides, providing a theoretical framework for their future medical development and application.

## 1 Introduction

**FIGURE 1:**
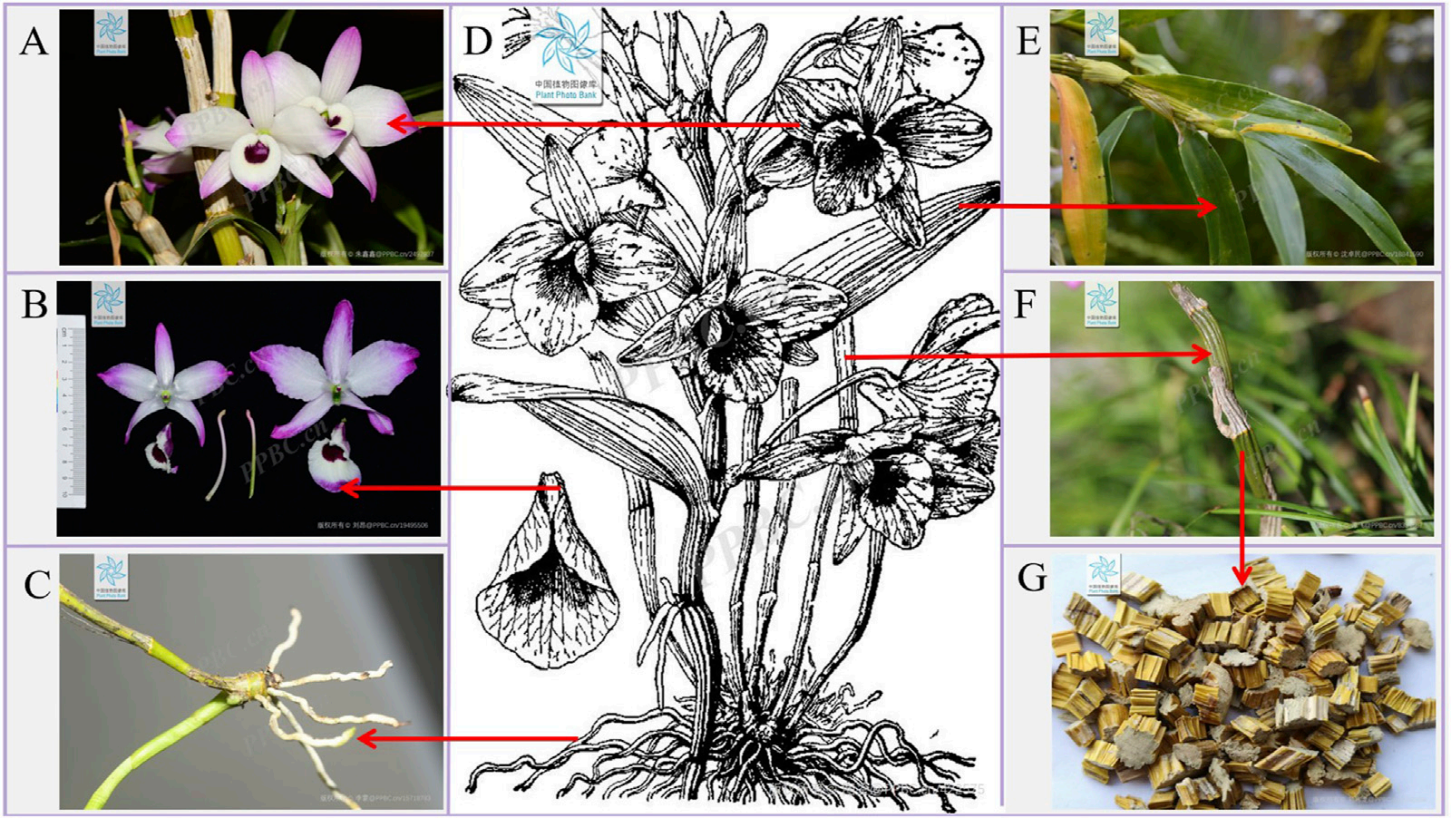
Plant morphology of *D. nobile*. **(A,B)** The flowers of *D. nobile*. **(C)** The roots of *D. nobile*. **(D)** The plant of *D. nobile*. **(E)** The leaves of *D. nobile*. **(F)** The rhizomes of *D. nobile*. **(G)** Dried rhizomes of *D. nobile* (The plant images in this article are from Plant Photo Bank of China, PPBC. https://ppbc.iplant.cn/).

Nature represents an abundant reservoir of botanical resources, with diverse climatic conditions, soil compositions, topographies, and landforms fostering a wide array of plant species. Among these, medicinal plants possess significant therapeutic potential, offering remedies for various ailments and contributing to human health and wellbeing (Yang et al., 2023). Within the Orchidaceae family, *Dendrobium* ranks among the three principal genera, encompassing over 1,602 recognized species worldwide (Plants of the World Online) (Wang, 2021a). Distributed extensively across Asia, more than 80 *Dendrobium* species have been documented in China (Zhai et al., 2022). *Dendrobium* species, such as *Dendrobium nobile* Lindl. (*D. nobile*), have been included in the Chinese Pharmacopoeia (2020 edition). Among them, there is relatively more research on *D. nobile*. Epiphytically growing on shady, humid trees or rocks under natural conditions, *D. nobile* has a fleshy and jointed stem, leathery leaves, and violet-hued flowers (Zhu et al., 2025) (Figure 1). Due to its prolific blooms and extended flowering period, it commands a significant place in the global cut flower industry, highlighting its dual purpose as an ornament and medicinal plant (Martin and Madassery, 2006).

With over two millennia of documented use in traditional Chinese medicine, *D. nobile* has been historically cited in classical Chinese texts (Hua et al., 2025). Clinically, it is commonly employed for the treatment or adjunct therapy of chronic pharyngitis, gastrointestinal disorders, ophthalmic conditions, diabetes, and cancer (Fan et al., 2023). In Bangladesh, it is also referred to as “Orchid” locally, and its pseudobulb extract is used for the treatment of eye infections and alleviate burns, and fresh leaf extract is used to put on fresh wounds for its healing properties (Hossain, 2009). Furthermore, *D. nobile* is also an important raw material in the development of various pharmaceuticals, such as Mailuoning injection, Shihuyeguang pills, and Shihumingmu pills (Wei et al., 2024). Aside from medicinal use, *D. nobile* is also a functional food; its juice, when consumed directly, is believed to quench thirst and provide cooling effects, particularly in summer. When soaked in water, it releases a pleasant aroma and offers long-term health benefits. Additionally, its extract possesses skin-whitening, moisturizing, and anti-aging activity, supporting its application in the cosmetics industry (Wang, 2021b).

Studying the chemical composition and structure of plants is a fundamental way to understand their biological activity. Extensive research has revealed that *D. nobile* contains a variety of bioactive compounds, including polysaccharides, alkaloids, phenols, and flavonoids (Liu et al., 2023; Zhang et al., 2023). *D. nobile* polysaccharides are an essential class of compounds that exhibit a broad spectrum of pharmacological activities such as anti-photoaging, improvement of complications of diabetes mellitus, ovarian protective, gastric protective, neuroprotective, anti-inflammatory and anti-viral effects (Lee et al., 2018; Pan et al., 2014; Wang H. et al., 2024). Advances in extraction and purification technologies have enabled the isolation of structurally diverse *D. nobile* polysaccharides, enriching the understanding of their structure–activity relationship relationships. The bioactivity of these polysaccharides is intricately linked to their structural parameters, including monosaccharide composition, molecular weight, glycosidic linkage patterns, and specific structural modifications. Unlike other small molecules, *D. nobile* polysaccharides have some unique advantages such as structural diversity, multi-function bioactivity, high chemical stability, and good biocompatibility, and therefore are receiving growing scientific interest.

Although *D. nobile* polysaccharides have been thoroughly studied, there are little reviews that summarize the current research progress. The systematic review of the article includes the extraction and purification methods, structural characteristics, biological activity, and structure-activity relationship of *D. nobile* polysaccharides, thus providing guidance to the future research directions and promoting the further usage of this polysaccharide.

## 2 Preparation strategies of *D. nobile* polysaccharides

Polysaccharides are normally extracted through a series of linked processes–extraction and purification–with neither of them being dispensable in attaining high-quality and purified products (Chen J. et al., 2024). The first important and crucial step is extraction; purification can determine the purity and functional integrity of the polysaccharides (Mohan et al., 2020; Xue et al., 2023). High-purity polysaccharides play a vital role in the research on molecular structure, bioactivity mechanisms, and potential applications.

### 2.1 Extraction of *D. nobile* polysaccharides

With continuous improvement of technology and ideas, it is becoming more intelligent, efficient, and environmentally friendly to obtain polysaccharides from plants. Selection of the appropriate technique is governed by the physicochemical properties of the target compounds, yield of polysaccharides, potential interfering substances, and overall process safety (Zhao et al., 2017). Comprehensively compare these parameters in different methods to determine the appropriate extraction method. Researchers also need to consider the impact on the environment, and efficient, green, economical, and environmentally friendly extraction methods are the most suitable.

**FIGURE 2:**
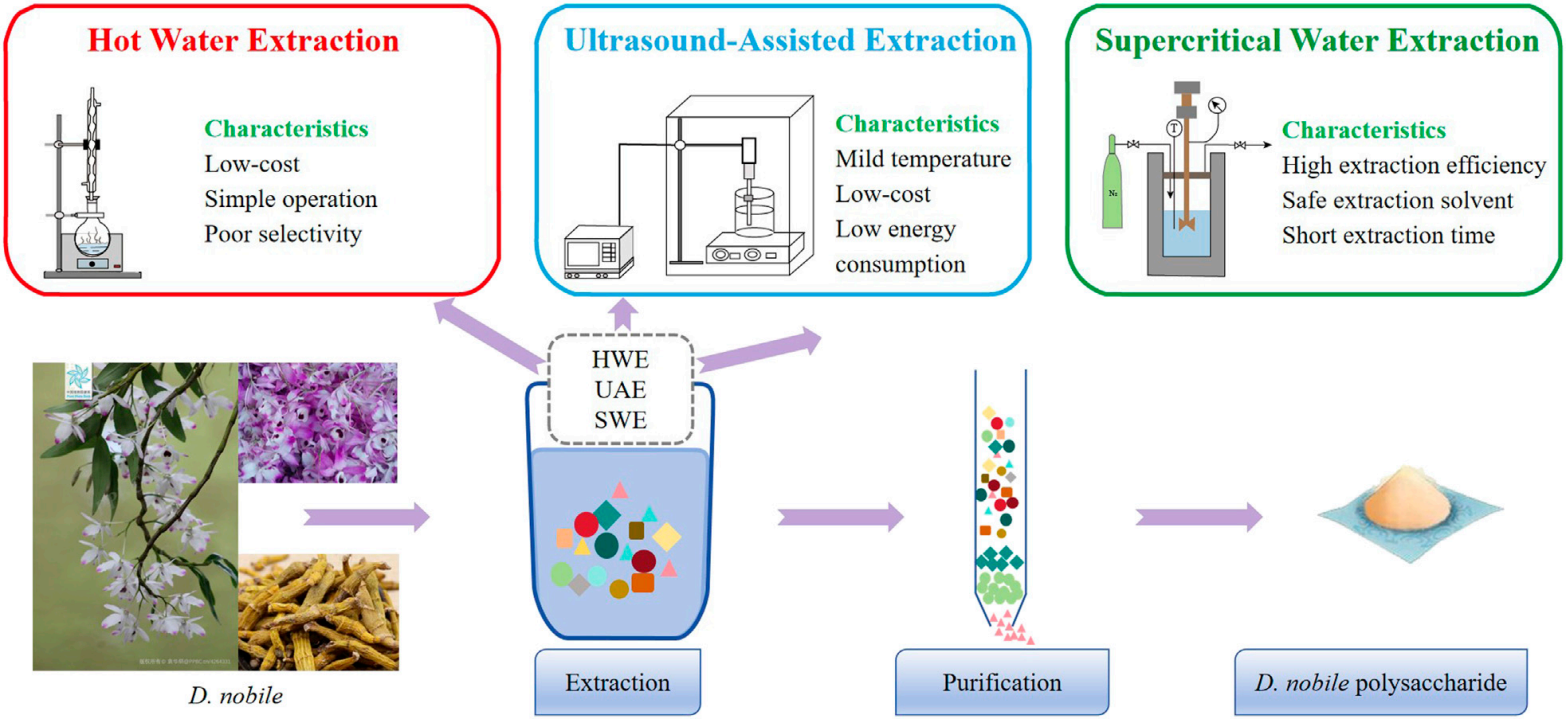
Regarding the extraction and purification of *D. nobile* polysaccharides (The plant images in this article are from Plant Photo Bank of China, PPBC. https://ppbc.iplant.cn/).

There are several things that should be determined before the polysaccharide’s extraction process begins. These include the processing methods of the raw materials, the extraction methods and conditions, the selection of solvents, as well as the final purification methods for the polysaccharides. *D. nobile* polysaccharides are mainly located within the cell walls. To facilitate their release, mechanical damage is usually inflicted on plant tissues. Additionally, lipophilic substances included in plants should be removed to minimize impurities to ease purification. The most used solvents are petroleum ether and ethyl acetate (Zhang et al., 2020). These procedures are the foundation for the subsequent process of extraction. The choice of extraction method plays a key role in determining the reliability and yield of subsequent experiments. Currently employed methods for isolating *D. nobile* polysaccharides include hot water extraction (HWE), ultrasound-assisted extraction (UAE), and subcritical water extraction (SWE) (Figure 2).

#### 2.1.1 Hot water extraction method

Polysaccharides as high-polarity macromolecules exhibit excellent solubility in hot water. Following the principle of “like dissolves like”, HWE is commonly adopted for their extraction (Chen et al., 2016). Jin et al. (2017) extracted *D. nobile* polysaccharides (JCS) using HWE with a solid-to-liquid ratio of 1:20 and a 4 h extraction cycle, repeated seven times to maximize yield. However, the broad solvency of water often results in the co-extraction of non-polysaccharide constituents, thereby complicating the purification process. To enhance extraction specificity and minimize contaminants, Luo et al. (2009) adopted a continuous experimental procedure. The raw material was first defatted with petroleum ether (60°C–90°C) for 2 h to eliminate lipid-soluble compounds, followed by an 80% ethanol wash for 2 h to remove oligosaccharides, glycosides, proteins, and other soluble impurities. Subsequent aqueous extraction was performed three times, each for 2 h. This multistep approach significantly improved polysaccharide purity and extraction efficiency while reducing interference from non-essential constituents.

#### 2.1.2 Ultrasound-assisted extraction method

UAE employs high-frequency sound waves (20–2,000 kHz) to induce cavitation, thereby disrupting plant cell matrices, enhancing solvent penetration, and accelerating mass transfer processes. The technique significantly facilitates intracellular polysaccharide release and is favored owing to its ease of operation, cost-effectiveness, and scalability in industrial settings (Cetinkaya et al., 2025) In the case of *D. nobile* polysaccharides (JCP) have been extracted via UAE using polyethylene glycol (PEG) as the solvent. Optimization through response surface methodology (RSM) identified optimal extraction conditions at 58.53°C, ultrasound power of 192.95 W, and PEG-200 concentration of 44.20% that yielded 15.57% of polysaccharide (Zhang et al., 2016). Essentially, the purpose of extraction is to get the maximum yield of polysaccharides without compromising their native structure and functional integrity.

#### 2.1.3 Subcritical water extraction method

SWE has become a promising and green method for extracting polysaccharides. SWE essentially reducing solvent residues and minimizing environmental impact by using water that is kept at high temperatures and pressures—below its critical point (Basak and Annapure, 2022). Using RSM, Liu et al. (2019) adjusted the SWE conditions for *D. nobile* polysaccharides. A polysaccharide recovery rate of 21.88% was obtained with an extraction temperature of 129.83°C, an extraction time of 16.71 min, and a pressure of 1.12 MPa, which resulted in a significant improvement in extraction efficiency (Liu et al., 2019).

### 2.2 Purification of *D. nobile* polysaccharides

Crude polysaccharides usually contain impurities such as pigments, proteins and low-molecular-weight compounds, which require further purification (Xue et al., 2024). Traditional methods of decolorization are hydrogen peroxide (H_2_O_2_) oxidation, adsorption on macroporous resin, and adsorption on activated carbon. When it comes to *D. nobile*, the most common two methods are the oxidation decolorization of H_2_O_2_ and activated carbon adsorption. Protein removal is typically achieved using the Sevag method or trichloroacetic acid precipitation, both of which operate by denaturing or precipitating proteins to separate them from polysaccharides (Li et al., 2019; Wang K. et al., 2024). Li et al. (2020) purified DNP (*D. nobile* polysaccharide) by using Sevag reagent to remove proteins from crude polysaccharides and decolorizing with activated carbon (Li et al., 2020). Further purification processes like column chromatography, membrane filtration, and stepwise precipitation are normally used to achieve an even higher purity (Liu et al., 2023; Wang et al., 2017). Columns of DEAE-cellulose, Sephadex G-series or Sepharose CL-series resins are commonly used among the chromatographic methods (Table 1). These processes yield highly purified *D. nobile* polysaccharide fractions suitable for omprehensive structural elucidation and bioactivity assessment.

**TABLE 1:** A summary of *D. nobile* polysaccharides extraction and purification methods.

Part	Extraction						Purification		Ref
	Polysaccharide fraction	Extraction methods	Time	Temperature and other parameters	Solid–liquid ratio	Polysaccharide yield (%)	Polysaccharide fraction	Purification methods	
Dried stems of *D. nobile*	DNP	HWE	2 h × 3	100°C	N/A	5%	N/A	N/A	Luo et al. (2009)
Dried stems of *D. nobile*	JCP	PEG-based UAE	N/A	58.53°C 192.95 W PEG-200 concentration of 44.20%	N/A	15.57%	N/A	N/A	Zhang et al. (2016)
Dried stems of *D. nobile*	JCS	HWE	4 h × 7	100°C	1:20	1.3%	JCS1	Q Sepharose Fast Flow column	Jin et al. (2017)
Dried stems of *D. nobile*	DNP-W	HWE	N/A	N/A	N/A	N/A	DNP-W4	DEAE-cellulose anion exchange and Sephacryl S-200 gel filtration chromatography	Wang et al. (2017)
Dried stems of *D. nobile*	*D. nobile* Lindl. polysaccharide	SWE	16.71 min	129.83°C, 1.12 MPa	1:25	21.88%	N/A	N/A	Liu et al. (2019)
*D. nobile*	DNP	HWE	3 h × 3	100°C	1:10	4.3%	DNPE6 (4), DNPE6 (11)	DEAE–Cellulose-52 anion-exchange column, Sephacryl S-200 and Sphadex G-100 columns	Li et al. (2019)
*D. nobile*	DNP	UAE	30 min	Ultrasonic power: 40 W	1:40	5.16% ± 0.41%	DNP1 and DNP2	DEAE-QFF and Sephacryl S-300 HR chromatography	Chen et al. (2022)
Dried ground stems of *D. nobile*	DN-P	HWE	6 h × 2	80°C	1:100	10.84%	F1, F2, F3, F4	Fractogel (BioSEC, Merck) column	Hsu et al. (2024)

Abbreviations: HWE, hot water extraction; PEG, polyethylene glycol; SWE, subcritical water extraction; UAE, ultrasound-assisted extraction; N/A, not available.

## 3 Structural characteristics of *D. nobile* polysaccharides

### 3.1 Molecular weight (Mw)

Mw as a physicochemical parameter has a determinant effect on the solubility, viscosity, and bioavailability of the polysaccharides (Wang et al., 2023). Gas-liquid permeation chromatography (GPC) and high-performance gel permeation chromatography (HPGPC) techniques are the two most frequently used to measure Mw with *D. nobile* polysaccharides (Li et al., 2024a; Zhang et al., 2020). In GPC, different elution times are attributed to polysaccharides with different Mw. A compound with known Mw is used as a reference standard to draw the calibration curve, and then the Mw of the unknown sample is determined by referring to the elution time. The current procedure has become the analytical method adopted in daily analyses. HPGPC is a combination of GPC with high-performance liquid chromatography (HPLC); shorter analysis time, better repeatability and ability to measure purity are achieved. Reported *D. nobile* polysaccharides Mw values are distributed within a broad range of 3.01–500 kDa, which can be explained by the sources of plants, method of extraction and purification, processing conditions (Li et al., 2020; Wang et al., 2017). Such Mw range is commonly construed to be the determinant behind such pronounced differences in pharmacological activity. Thus, Mw continues to be an essential parameter during both basic and applied research of polysaccharide.

### 3.2 Monosaccharide composition

Polysaccharides are polymers of monosaccharides with the exact composition significantly affecting functional properties. Examples of common monosaccharides are glucose (Glc), galactose (Gal), arabinose (Ara), and fucose (Fuc). The composition is usually identified by 1-phenyl-3-methyl-5-pyrazolone (PMP) derivatization technique or by acetylation-based technique (Liu et al., 2021). The polysaccharides of *D. nobile* are structurally diverse and often contain Ara, Gal, Glc, Man, Xyl, Rha and GalA (Hu et al., 2024; Li et al., 2022) (Table 2). The most commonly found monosaccharide are Gal, Glc and Man. In spite of the fact that certain polysaccharides contain identical monosaccharide, variation in molar proportion endows them with distinct structural and functional properties. As an example, both DNP and DNLP contain Man, Glc, and Gal, but the molar ratios of these components, 12.49%:65.2%:6.4% and 26.9%:66.2%:6.9%, indicate the differences in composition that can be the basis of the variations in bioactivity (Hu et al., 2024; Zhang et al., 2020).

**TABLE 2:** Source, compound name, molecular weights, monosaccharide composition, structures of *D. nobile* polysaccharides, and analytical techniques.

Source	Compound name	Molecular weights	Monosaccharide composition	Structures	Analytical techniques	Ref
Dried stems of *D. nobile*	DNP	87.6 kDa	Rha: Ara: Xyl: Man: Glc: Gal = 1:2.8:2.2:30.8:117.9:31.8	The backbone of DNP was composed of (1→6)-linked-*α*-d-glucopyranosyl, and (1→6)-linked-*α*-d-galactopyranosyl residues, with branches of (1→4)-linked-*α*-d-glucopyranosyl and (1→4)-linked-*α*-d-mannopyranosyl residues	GC–MS, GPC, IR, NMR	Luo et al. (2009)
Dried stems of *D. nobile*	JCS1	23 kDa	Glc: Man: Xyl: Ara = 40.2:2.0:1.3:1.0	The backbone of JCS1 might consist of repeated 1,4-linked *β*-Man*p* and 1,4-linked *α*-Glc*p* units with branches at the C-6 of 1,4-linked *α*-Glc*p* substituted by 1,4-linked *α*-Xyl*p* and T-*α*-Ara*f* linked at C-4 of 1,4-linked *α*-Xyl*p*.The other branches might be linked by T-*α*-Glc*p* at C-6 of 1,4-linked *α*-Glc*p*	HPGPC, GC, IR, FT-IR, NMR	Jin et al. (2017)
Dried stems of *D. nobile*	DNP-W4	500 kDa	Man: Glc: Gal: Xyl: Rha: GalA = 1.0: 4.9: 2.5: 0.5: 1.0: 0.9	DNP-W4 possesses a backbone of (1→4)-linked *β*-d-Glc*p*, (1→6)-linked *β*-d-Glc*p*, and (1→6)-linked *β*-d-Gal*p*, with substitutes at O-4/6 of Glc*p* residues and O-3 of Gal*p*. The side chains may be composed of terminal Man*p*, (1→6)-linked *β*-d-Manp, (1→3)-linked *β*-d-Glcp, *β*-d-Glc*p*, (1→4)-linked *α*-d-GalA*p*, (1→2)-linked *α*-l-Rha*p*, and Xyl*p*	HPGPC, GC-MS, FT-IR, IR, NMR	Wang et al. (2017)
Dried stems of *D. nobile*	*D. nobile* Lindl. polysaccharide	85.72 kDa	Ara: Gal: Glc: Man = 1.05: 1.6: 48.63: 48.73	N/A	N/A	Liu et al. (2019)
*D. nobile*	DNPE6(4)	99.2 kDa	Ara: Glc: Gal: Man = 2.5: 0.9: 0.3: 0.8	The linkage type of DNP were →1)-l-Ara*f*-(3→, →1)-d-Glc*p*-(4→, →1)-d-Gal*p*-(3→, →1)-d-Gal*p*-(6→, →1)-d-Man*p*-(3, 6→, and T-d-Man*p*	UV, FT-IR, HPGPC	Li et al. (2019)
*D. nobile*	DNPE6 (11)	3.01 kDa	Man: Glc: Gal = 3: 11: 3	The backbone of DNPE6(11) was composed of [→6)-d-Man*p*-(1→4)-d-Glc*p*-(1→]	HPGPC, UV and FTIR	Li et al. (2020)
*D. nobile*	DNLP	1.2–11.2 kDa	Man: Glc: Gal = 26.9%: 66.2%: 6.9%	N/A	N/A	Zhang et al. (2020)
*D. nobile*	DNP1	67.72 kDa	Man: Glc = 75.86 ± 0.05%: 24.14% ± 0.05%	DNP1 is a straight-chain glucomannan composed mainly of *β*-1,4-D-Manp, *β*-1,4-day-Glc*p* residues, and some acetyl groups linked to C-2 or C-3 of the mannose residues	FT-IR, and NMR	Chen et al. (2022), Li et al. (2024)
*D. nobile*	DNP2	37.45 kDa	Man: Glc = 72.32 ± 0.03%: 27.68% ± 0.03%	N/A	N/A	Chen et al. (2022)
*D. nobile*	DNLP	N/A	Rha: Ara: Xyl: Man: Glc: Gal = 1.00: 1.65: 2.58: 1.08: 1.83: 1.15	N/A	N/A	Li et al. (2022)
*D. nobile*	DNP	106.6 kDa	Man: Glc: Gal = 23.2%: 68.5%: 3.5%	N/A	N/A	Luo et al. (2022)
*D. nobile*	DNP	13.2 kDa	Man: Glc: Gal = 12.49%: 65.2%: 6.4%	N/A	N/A	Hu et al. (2024)

Abbreviations: NA, not available.

### 3.3 Chemical structures

**FIGURE 3:**
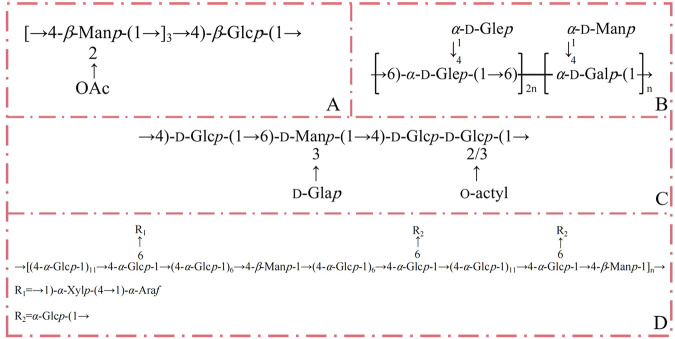
The hypothetical structure of *D. nobile* polysaccharides **(A)** DNP1. **(B)** DNP. **(C)** DNPE6 (11). **(D)** JCS1.

Numerous studies have elucidated the chemical structure of polysaccharides derived from *D. nobile*. A novel polysaccharide, DNP1, was isolated, with its core structure consisting of →4)-*β*-Man*p*-(→1 and →4)-*β*-Glc*p*-(→1 sugar residues forming the backbone. Nuclear magnetic resonance (NMR) spectroscopy revealed that the repeating units of DNP1 likely consist of [→4)-2-OAc-*β*-Man*p*-(1→]3→4)-*β*-Glc*p*-(1→) (Li et al., 2024b). Luo et al. (2009) isolated a heteropolysaccharide, DNP, from the dry stems of *D. nobile*. Structural analysis through gas chromatography-mass spectrometry (GC-MS), infrared spectroscopy (IR), and NMR confirmed that DNP comprises (1→6)-linked-*α*-d-glucopyranosyl and (1→6)-linked-*α*-d-galactopyranosyl units, along with (1→4)-linked-*α*-d-glucopyranosyl and (1→4)-linked-*α*-d-mannopyranosyl branches (Luo et al., 2009). The proposed structure of DNP is depicted in Figure 3. Additionally, a study suggests that the polysaccharide extracted from *D. nobile* is mannoglucan. Jin et al. (2017) characterized polysaccharide JCS1 using chemical and spectral methods. The structure of JCS1 is primarily composed of *α*- and *β*-glycosidic linkages, with 1,4-linked Man and Glc as the main chains. Branching structures, formed through specific linkages, are present along the chains. The backbone of JCS1 is hypothesized to consist of alternating 1,4-glycosidic bonds between *β*-Man*p* and *α*-Glcp units, with branching at the C-6 position of the 1,4-linked *α*-Glc*p* residues, where 1,4-linked *α*-Xyl*p* units replace *α*-Glc*p*. A T-*α*-Ara*f* is attached at the C-4 position of the 1,4-linked *α*-Xyl*p*, and additional branches may be formed via T-*α*-Glc*p* linkages at C-6 of the 1,4-linked *α*-Glc*p*. These findings support the classification of JCS1 as a mannoglucan (Jin et al., 2017). Research of *D. nobile* polysaccharides is primarily focused on the linkage, branching, and methylation structure of the monosaccharide units. Chemical composition of the polysaccharides has been presented in Table 2. Simple structure of polysaccharides is made up of monosaccharides linked to each other by glycosidic linkages, differing in the proportion of each monosaccharide. Such structural diversity makes analysis of the chemical composition of *D. nobile* polysaccharides very inconvenient.

## 4 Biological activity of *D. nobile* polysaccharides

### 4.1 Anti-photoaging effect

#### 4.1.1 Anti-UVA and UVB effect

The skin acts as a shield and protects the internal parts of the body against the external environmental factors. The skin, especially due to its large surface area and constant exposure to the environment, is especially vulnerable to the effects of ultraviolet (UV) radiation (Zhang M. et al., 2024). UV radiation is the most immediate physical environmental factor that comes in contact with the skin and is greatly linked to various skin disorders. As much as mild exposure to UV rays is beneficial, overexposure has adverse effects, which culminate to skin damages (Wang Z. et al., 2024). Chronic UV-induced exposure could be in the form of sunburn, immune suppression, malignancy and photoaging. Skin aging is categorized into the endogenous and exogenous with UV radiation as the main exogenous aging factor, also referred to as the photoaging (Dusabimana et al., 2024; Li et al., 2024). The UV spectrum (100–400 nm) is divided into three bands: UVA (320–400 nm), UVB (280–320 nm), and UVC (100–280 nm). While UVC has a potent cellular-killing effect, it is almost entirely absorbed by the ozone layer, posing minimal risk to human health. Therefore, UVA and UVB are the principal contributors to skin damage (Chen W. et al., 2024).

**FIGURE 4:**
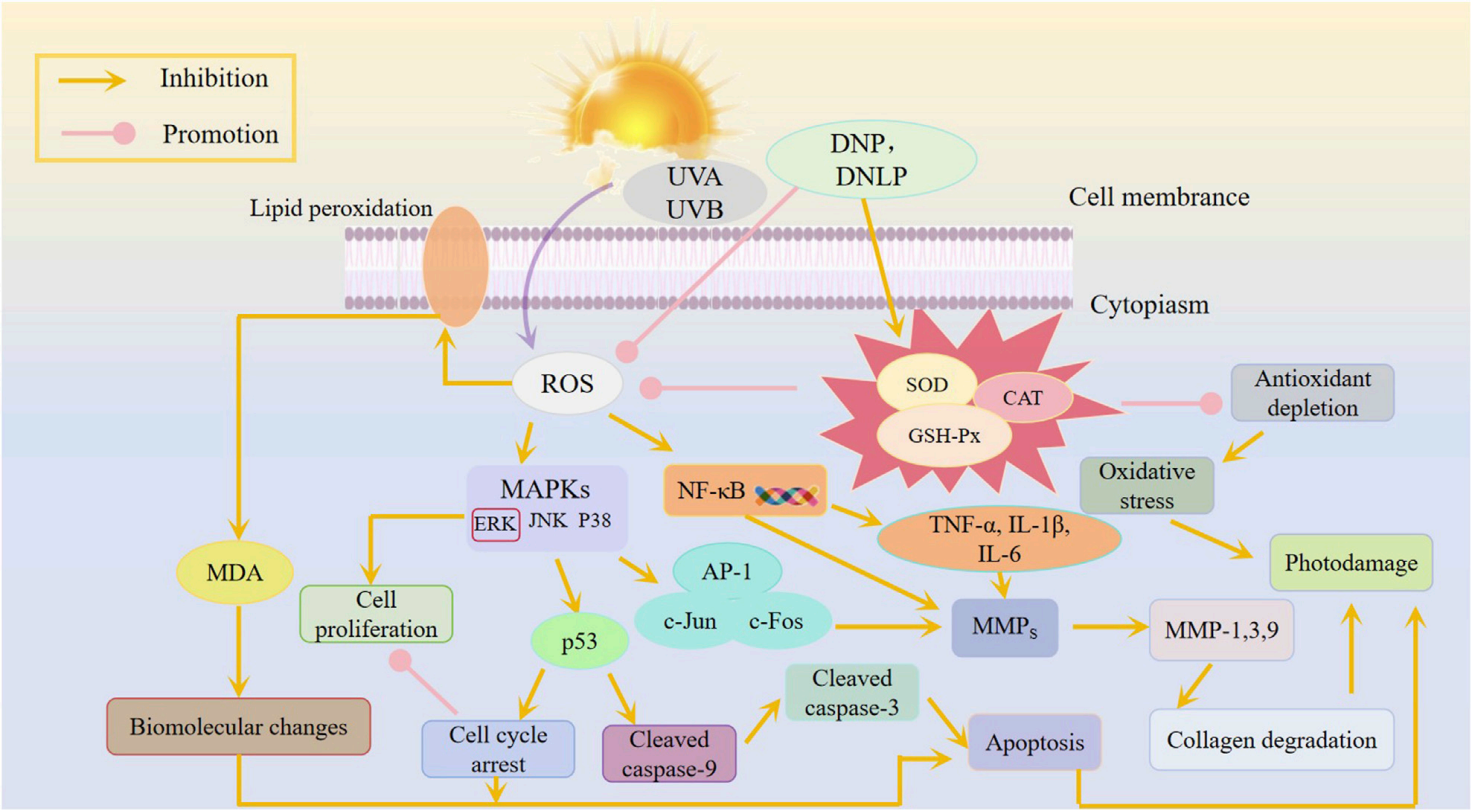
Potential mechanism of polysaccharides from *D. nobile* in anti-UVA and UVB.

Polysaccharides derived from *D. nobile* exhibit anti-photoaging effects against both UVA and UVB radiation (Table 3). Long et al. (2023a) established a mouse model of UVB-induced acute photoaging and evaluated the anti-photoaging effects of varying doses of DNP (50, 200 mg/mL), with vitamin E serving as a positive control. UV radiation induces reactive oxygen species (ROS) that trigger oxidative stress, accelerating skin aging and wrinkle formation. DNP can reverse UVB-induced oxidative stress by enhancing the activity of antioxidant enzymes (SOD, CAT, GSH-Px) and reducing MDA levels. UVB irradiation also activates inflammatory cells, leading to inflammation. Excessive UVB irradiation can lead to a large accumulation of intracellular ROS, thereby promoting the activation of AP-1 and NF -κB, inducing abnormal secretion of pro-inflammatory cytokines such as TNF-*α*, IL-1*β*, and IL-6, and stimulating the transcription of MMPs genes (Cui et al., 2022). TNF-*α* and IL-1*β* are the main cytokines in the inflammatory response, which can stimulate the production of secondary inflammatory mediators, induce the migration of inflammatory cells, and trigger the inflammatory response (Xiao et al., 2022). Compared to other pretreatment groups, the UVB + DNP-H group showed a significant reduction in IL-1*β* expression (*P* < 0.05). In the current study, DNP was found to have inhibitory action on expression of IL-6 and TNF-*α*. UV radiation activates matrix metalloproteinases (MMPs) which cleave collagen and elastin and increases skin photoaging. UVB + DNP-H and UVB + DNP-L groups inhibited the UVB-induced upregulation of the MMP, thus protecting extracellular matrix (ECM) components, and maintaining the integrity of collagen and elastin fibers. These findings were supported by Masson staining and quantification of hydroxyproline (HYP) contents. Such findings provide a scientific basis of DNP in the alleviation of UVB-induced skin photoaging (Long et al., 2023b; Wang et al., 2021b). Besides, the team of researchers evaluated the effect of DNP on UVB-induced oxidative stress and apoptosis in HaCaT cells. It was found out that DNP significantly inhibited the reduction of the viability and proliferation of the HaCaT cells induced by UVB treatment. DNP scavenged ROS increased endogenous antioxidant enzyme activity, reduced MDA levels, and partially alleviated cell cycle arrest, demonstrating its antioxidant and anti-apoptotic properties. The Western blot analysis revealed that DNP suppresses UVB-induced oxidative injury and apoptosis in HaCaT cells by regulating mitogen-activated protein kinase (MAPK) signaling pathways (Long et al., 2023a; Zhai et al., 2022). Li et al. (2022) also examined whether *D. nobile* polysaccharides (DNL) have any effect on photoaging caused by UVA *in vitro* in human foreskin fibroblasts (HFF-1). The results showed that DNL polysaccharides significantly reversed UVA-induced HFF-1 cell damage, improved oxidative stress, and regulated ROS levels, as well as SOD, CAT, and GSH-Px activity. Additionally, DNL polysaccharides reduced S-*β*-Gal expression and mitigated UVA-induced photoaging by inhibiting the secretion of MMP-1, MMP-2, MMP-3, and MMP-9 (Li et al., 2022; Yang et al., 2023). In conclusion, *D. nobile* polysaccharides effectively combat UVA and UVB-induced photoaging through their antioxidant, anti-inflammatory, and anti-apoptotic properties (Figure 4).

**TABLE 3:** Summary of biological activities of *D. nobile* polysaccharides.

Biological activities	Polysaccharide name	Types	Testing subjects	Doses/duration	Effects/mechanisms	Ref
Anti-UVA and UVB effect	DNP	*In vivo*	Kunming mice	50 mg/mL, 200 mg/mL	DNP can reverse UVB induced oxidative stress by increasing the activity of antioxidant enzymes and reducing MDA content. Compared with other pretreatment groups, the expression level of IL-1β, IL-6 and TNF-α were decreased in the UVB + DNP-H and UVB + DNP-L group. In addition, both groups were able to reduce the increase in MMPs expression induced by UVB radiation	Long et al. (2023b)
	DNP	*In vitro*	HaCaT cells	200 μg/mL, 800 μg/mL	DNP improves UVB induced oxidative damage and apoptosis in HaCaT cells by regulating MAPKs	Long et al. (2023a)
	DNLP	*In vitro*	HFF-1 cells	0.06 mg/mL, 0.18 mg/mL, 0.54 mg/mL	DNLP could significantly reverse UVA induced HFF-1 cell damage, improve its oxidative stress state, regulate ROS content, and levels of SOD, CAT, and GSH-Px. In addition, DNLP improves UVA induced photoaging mediated collagen degradation by inhibiting the secretion of MMP-1, MMP-2, MMP-3, and MMP-9 protein expression, reducing the phosphorylation activation of the JNK/c-Fos/c-Jun pathway, and decreasing the expression of SA-*β*-Gal	Li et al. (2022)
Anti-blue light effect	DN-P and DN-PP	*In vitro* and *in vivo*	ARPE-19 cells 661W cells, and *Drosophila*	DNP: 50, 100, 200 μg/mL. DN-PP: 25, 50, 100 μg/mL	Pretreatment with polysaccharides from *D. nobile* can reduce ROS and superoxide levels, as well as decrease the levels of antioxidant enzymes (SOD1 and CAT), inhibit the increase of IL-1β and IL-6, and alleviate eye damage in *drosophila* under blue light irradiation. It can also increase the expression levels of *ninaE, norpA, Gαq, Gβ76C, Gγ30A, Trp,and Trpl*	Hsu et al. (2024)
Ovarian protective effect	DNLP	*In vivo*	Female Sprague-Dawley rats	200 mg/kg/day	Compared with the model group, the body weight of PCOS rats treated with DNLP was reduced (*P* < 0.01), the estrous cycle returned to normal, and serum testosterone levels and insulin resistance were also reduced. In addition, the volume of the corpus luteum increases, the number of antral follicles decreases, and the thickness of the granulosa cell layer increases. DNLP treatment also increased the expression levels of PCNA, and inhibited cell apoptosis in PCOS ovarian tissue by regulating apoptosis related proteins Bax, Bcl-2, and caspase-3	Zhang et al. (2020)
	DNP	*In vivo*	Female Sprague-Dawley rats	200 mg/kg/day	Administration of DNP significantly reduced blood glucose, serum insulin, and HOMA-IR levels in PCOS rats, and restored the expression of IGF1 and IGF1R. Besides, DNP intervention can also promote GCs glycolysis and improve the mechanism of follicular dysplasia. Importantly, SIRT2 may be a key factor in regulating the glycolysis rate of granulosa cells by DNP.	Hu et al. (2024)
Testicular protective effect	DNLP	*In vivo*	Male Sprague‐Dawley rats	400 mg/kg/day	DNLP plays a protective role in DM induced reproductive damage by regulating the expression of SIRT1 in testicular tissue	Lei et al. (2022)
	DNP	*In vivo*	Male C57BL/6J mice	200 and 400 mg/kg	DNP can improve spermatogenesis in streptozotocin induced diabetes mice by regulating glycolysis pathway	Luo et al. (2022)
Gastric protective effect	JCP	*In vivo*	Male Wistar rats	L-JCP: 100 mg/kg. H-JCP: 300 mg/kg	Compared with the vehicle group, JCP treatment showed a decrease in inflammatory cells, reduced bleeding, and effectively increased the expression level of SOD in rat gastric tissue, while reducing MDA content. In addition, oral administration of 300 mg/kg JCP to rats significantly increased EGF production. H-JCP can reduce the expression levels of gastric p-JNK, p-ERK, MMP-2, and MMP-9	Zhang et al. (2018)
Neuroprotective effect	DNP	*In vivo*	Male Sprague-Dawley rats	100 mg/kg/day	The mitochondrial membrane and cristae in the DNP group improved compared to the VD group. Compared with the model group, the expression of GSH, xCT, and GPx4 in the hippocampus of the DNP group was significantly upregulated (*P* < 0.01). TEM results showed that the DNP group had an increase in synaptic vehicles, a significant increase in synaptic active zone (SAZ) length and PSD thickness, and a significant upregulation of PSD-95 protein expression	Ming et al. (2023)
	DNP	*In vivo*	Female Sprague-Dawley rats	100 mg/kg/day	After DNP treatment, there was improvement and a decrease in iron content (24 and 48 h after injury). The HE staining after 28 days of treatment showed that the spinal cord tissue defect area in the DNP group was smaller compared to the model group. And compared with the sham operation group, the xCT, GSH, Gpx4, and GRSFI in the spinal cord tissue of the model group rats decreased (*P* < 0.05), while the expression of these indicators increased after DNP treatment. DNP treatment increased NeuN^+^ cells at 14 and 28 days after SCI.	Huang J. et al. (2024)
Anti-inflammatory effect	DNP1 and DNP2	*In vitro*	RAW264.7 macrophages	12.5, 25, 50, 100, 200 μg/mL	DNP1 and DNP2 significantly reduce the production of inflammatory factors in a dose-dependent manner. DNP1 may exert immunomodulatory effects by binding to the TLR4-MD2 complex and inhibiting the TLR4-MD2 mediated signaling pathway	Chen et al. (2022), Li et al. (2024)
Anti-viral effect	DNPE6 (4)	*In vivo*	*Nicotiana tabacum* cv. K326	125 μg/mL	The protective activities of DNPE6 (4) against CMV and TMV were 40.4% and 69.9%, respectively. After DNPE6 (4) induction, NADPH oxidase and NAD(P)H increased. In addition, DNPE6 (4) can stimulate the activity of defensive enzymes (POD, PAL, SOD) in tobacco to resist plant virus infection. DNPE6 (4) could increase the expression level of SOD and the expression levels of ICSI, EDSI, and PRI up-stream and down-stream of the Salicylic acid (SA) pathway, while inhibiting the protein expression of CAT.	Li et al. (2019)
	DNPE6 (11)	*In vivo*	*Nicotiana tabacum* cv. K326	500 μg/mL	DNPE6 (11) has significant therapeutic and inactivation activity against CMV, significant protective effect against PVY. In addition, preliminary mechanistic studies have found that DNPE6 (11) has a strong binding ability to the coat protein of cucumber mosaic virus	Li et al. (2020)

#### 4.1.2 Anti-blue light effect

Blue light, primarily emitted by the sun, offers various health benefits, including regulating human behavior and circadian rhythms, enhancing memory and cognitive functions, and stabilizing emotions. However, prolonged exposure to blue light can lead to eye damage due to its high energy (Chan et al., 2023). Consequently, excessive exposure to blue light should be avoided. With the increasing use of electronic devices, prolonged exposure to blue light has become inevitable. Despite the rising prevalence of protective measures, long-term research is essential in the development of more effective approaches to protect the eyes against the dangers of blue light (Li et al., 2024; Liu et al., 2024). *D. nobile*-derived polysaccharides, namely DNP and DN-PP, have significant protective effects against retinal damage by blue light. When cell population of ARPE-19 and 661W cells were pre-incubated with DNP and DN-PP in various concentrations, cellular viability increased significantly compared to that of cells exposed only to blue light in cell viability assays. Constant exposure to blue light often leads to the buildup of ROS and the development of an oxidative state in the retina (Ouyang et al., 2020; Yan et al., 2025). Blue light exposure notably increased ROS and superoxide levels in 661W cells, while ARPE-19 cells showed a lower, less sensitive response. A dose-dependent decrease in the ROS levels of 661W was noticed through pre-treatment with different doses of DNP, DN-PP, and the positive control of alpha-lipoic acid (ALA). In both 661W and ARPE-19 cells, the production of superoxide also reduced compared to blue light-treated cells. Moreover, the increased expression of IL-1 and IL-6 by the blue light was inhibited in a dose-dependent effect by pretreatment with DNP and DN-PP and reduced the increase in SOD1 and CAT antioxidant enzymes following blue light exposure. Opsins, G-protein-coupled receptors involved in photosensitivity, were significantly upregulated by blue light. In 661W cells, DN-P (400 μg/mL) and DN-PP (100 μg/mL) reversed the expression of opsin 3 to levels similar to the control group. In ARPE-19 cells, only DN-PP (100 μg/mL) showed comparable levels to the control group. Furthermore, the protective mechanism of DN-P and DN-PP against blue light damage *in vivo* was investigated using *Drosophila*. Electroretinography (ERG) analysis demonstrated that DN-P (12.5 mg/mL) exerted neuroprotective effects on blue light-induced retinal injury in *Drosophila*. q-PCR analysis revealed that DN-P (12.5 mg/mL) pretreatment restored the expression levels of key light-responsive genes (*ninaE*, *norpA*, *Gαq*, *Gβ76C*, *Gγ30A*, *Trp*, and *Trpl*) in *Drosophila*. Compared to the blue light-only group, the DN-P (12.5 mg/mL) group exhibited increased expression levels of these genes. In conclusion, pretreatment with DNP provides protection for retinal cells and photoreceptors against blue light-induced damage (Chen W. et al., 2024; Hsu et al., 2024).

### 4.2 Improvement of complications of diabetes mellitus

#### 4.2.1 Testicular protective effect

Diabetes mellitus (DM) is a metabolic illness that is characterized by chronic hyperglycemia. Though DM is not necessarily lethal, persistent increases in blood sugar level can trigger malfunction or deterioration of several organs and systems leading to a variety of complications (Wang K. et al., 2024). One of these complications is the negative impact of DM on male reproductive system which may decrease sperm number and quality, testicular dysfunction and inhibit sperm production and maturation, and eventually influence male fertility (Xu et al., 2024). In this regard, DNP has been proven to provide a protective influence on the testicular tissue in DM. In the study by Lei et al. (2022), SD male rats were separated into four groups: nondiabetic, diabetic, model + metformin and model + DNLP. Diabetes was developed through a high-fat diet and intraperitoneal injection of 35 mg/kg streptozotocin (STZ). Major predictors of male reproductive health include testicular weight, testosterone concentration, sperm volume and sperm motility (Huang R. et al., 2024). Compared to the control group, rats in the model group exhibited significant reductions in testicular and epididymal quality (*P* < 0.05), as well as decreased sperm count and motility (*P* < 0.05). On the other hand, the DNLP improved significantly all four indicators in the rats (*P* < 0.05). The seminiferous tubules in the model group were highly atrophic with a sparse spermatogenic cells arrangement and had a very low sperm count. DNLP improved these pathological changes and promoted the quantity of spermatogonia and spermatocytes. Quantitative evaluation of apoptotic cells demonstrated that the apoptotic process in the testicular tissue in the DNLP group decreased significantly as compared to the model group (model + DNLP group: 43.13 ± 7.21; model group: 70.67 ± 3.16, *P* < 0.05). DNLP treatment also increased the expression of PCNA which is a basic protein of cell proliferation. Male reproductive health also relies on such a key regulator of glucose metabolism SIRT1, with reduced SIRT1 expression having been reported to contribute to germ-cell apoptosis in diabetic mice. DNLP promoted the level of mRNA and SIRT1 protein in DM rats (Lei et al., 2022; Nie et al., 2020). The present findings indicate that DNLP can reduce the DM-induced reproductive harm through the regulation of SIRT1 in testicular tissue expression, but the mechanisms underlying DM-induced tissue damage should be further investigated.

Thereafter, the investigators determined the protective role of another polysaccharides in *D*. *nobile* (DNP) on diabetic mellitus (DM)-induced reproductive dysfunction. In this experiment, the major concern was to assess the effect of DNP on glucose homeostasis in mice with DM focusing on the glycolysis pathway. The results showed that DNP could improve the spermatogenic dysfunction induced by DM by improving the abnormal structure of testis, inhibiting the apoptosis of spermatogenic cells and promoting proliferation. DNP also recovered the architecture and physiologic role of sertoli cells (SC) in DM mice, increased the expression of SC marker GATA4, WT1, and vimentin and the expression of major glycolysis-limiting enzymes LDHA, PKM2, and HK2. On the whole, these results indicate that DNP enhances spermatogenesis in streptozotocin-induced diabetic mice through the regulation of glycolysis pathway (Fan et al., 2023; Luo et al., 2022).

#### 4.2.2 Improve retinal inflammation

**FIGURE 5:**
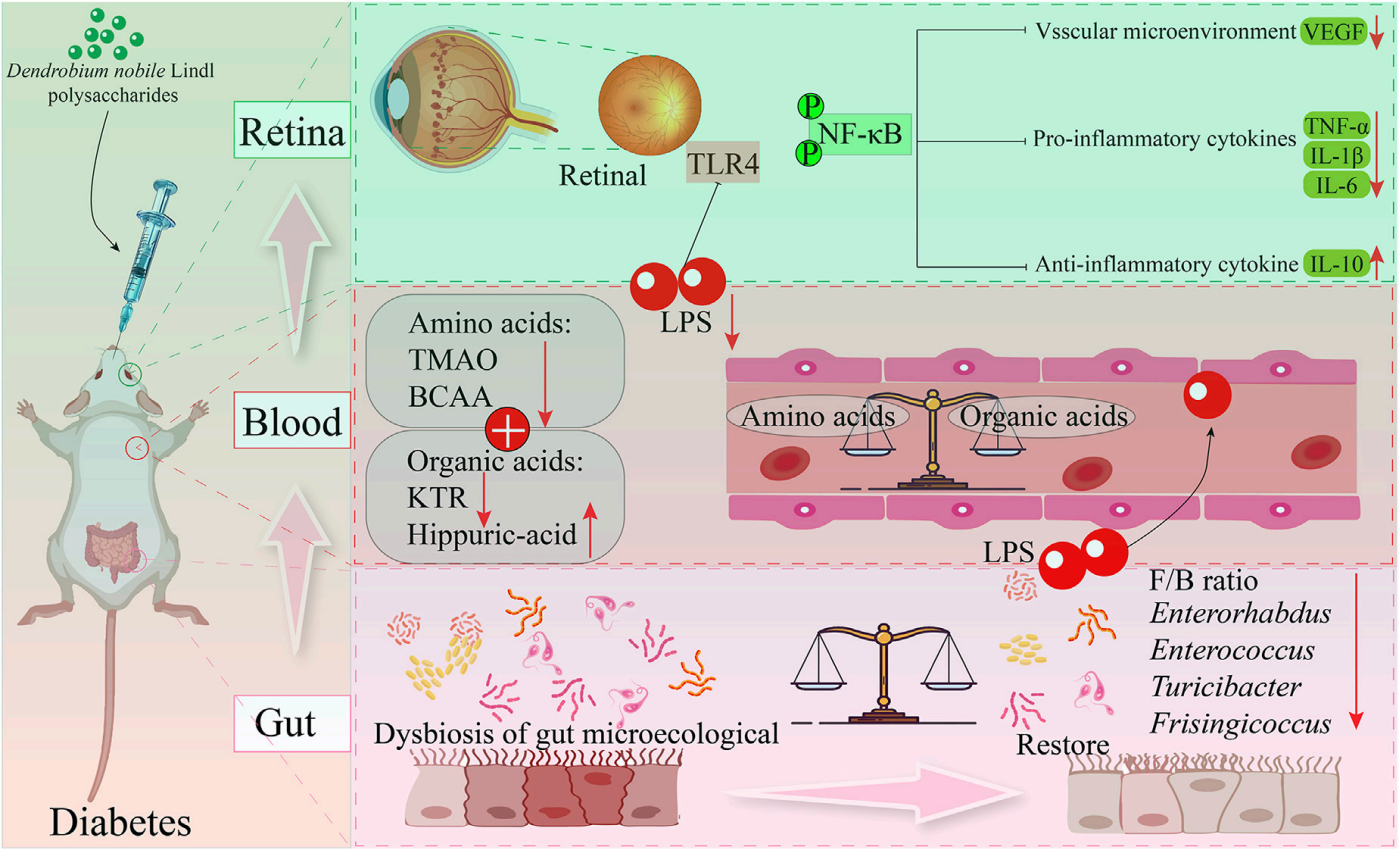
Potential mechanism of polysaccharides from *D. nobile* in improve retinal inflammation.

Retinopathy is one of the most common complications in patients with DM (Cai et al., 2025). *D. nobile* polysaccharides can improve the inflammatory microenvironment in the retina of DM rat models. This study investigated the effects of DNP (*D. nobile* polysaccharides) at doses of 50, 100, and 200 mg/kg on the inflammatory microenvironment in DM rats, exploring the potential mechanisms through intestinal microbiology, metabonomic, and transcriptomics. Following DNP treatment, typical DM symptoms were alleviated, and the imbalance in the gut microbiota was reversed. DNP treatment increased the abundance of *Parabacteroides* and reduced the levels of *Enterorhabdus*, *Enterococcus*, *Turicibacter*, and *Frisingicoccus*. Dysbiosis of the gut microbiota affects the secretion of metabolites, and after DNP treatment, the types and levels of metabolites in DM rats closely resembled those in the control group, while untreated DM rats showed marked changes. This dysbiosis and the alterations in metabolite profiles can compromise intestinal barrier function (Chen et al., 2023). H&E staining of colon tissue from the DM rat model revealed that DNP treatment reduced mucosal degeneration and necrosis in the colon and partially restored the structure of intestinal glands. Additionally, the expression of ZO-1 and claudin-1 proteins was increased. DNP treatment also significantly reduced serum LPS levels, a marker of endotoxemia, and decreased the metabolism of intestinal and circulating branched-chain amino acids (BCAAs). Conversely, the metabolism of intestinal and circulating hippurate was increased, which inhibited the TLR4/NF-κB signaling pathway and ultimately improved the retinal inflammatory microenvironment. This multi-omics study is the first to systematically evaluate the therapeutic effect of DNP on type 2 DM and its associated retinal inflammation (Wang R. et al., 2025) (Figure 5). Based on these findings, DNP treatment presents a promising strategy for preventing or treating DM-related complications.

### 4.3 Ovarian protective effect

Polycystic ovary syndrome (PCOS) is the most common endocrine illness that affects women of reproductive age, and most often it is caused by abnormalities of ovarian follicles, particularly of the follicular membrane (Helvaci and Yildiz, 2025). Symptoms that characterize the condition include excessive hair growth, acne, and obesity (Mousa et al., 2024). In order to test the therapeutic value of *D. nobile* polysaccharides (DNLP) against PCOS, researchers performed animal studies. PCOS was induced by the daily intraperitoneal treatment of the rats with letrozole (1 mg/kg) in a solution of carboxymethyl cellulose (0.5%) and feeding them a high-fat diet (30 days). Therapeutic effect of the oral DNLP administration (200 mg/kg/day) was then evaluated for 21 days according to quantitative analysis of ovarian morphology, serum glucose, insulin, hormone levels, expression of proliferating cell nuclear antigen (PCNA), Bax, Bcl-2, caspase-3 mRNA in ovarian tissue, and ovarian granulosa cell apoptosis. As compared with the model group, DNLP treatment led to a statistically significant decrease in body weight (*P* < 0.01) and normalized the estrous cycle. Even though there was a reduction in serum testosterone levels and indicators of insulin resistance, there was no change in the level of luteinizing hormone. A histological examination showed a significant decrease in follicles and corpus luteum, an increase in antral follicles, a disorganized arrangement of granulosa cells, and a thinning of the granulosa cell layer in the model group. After DNLP treatment the volume of corpus luteum was enhanced, the amount of antral follicles was reduced, and the layer of granulosa cells thickened when compared with the model group. The overall result of these studies suggests that DNLP has positive therapeutic in the treatment of PCOS.

**FIGURE 6:**
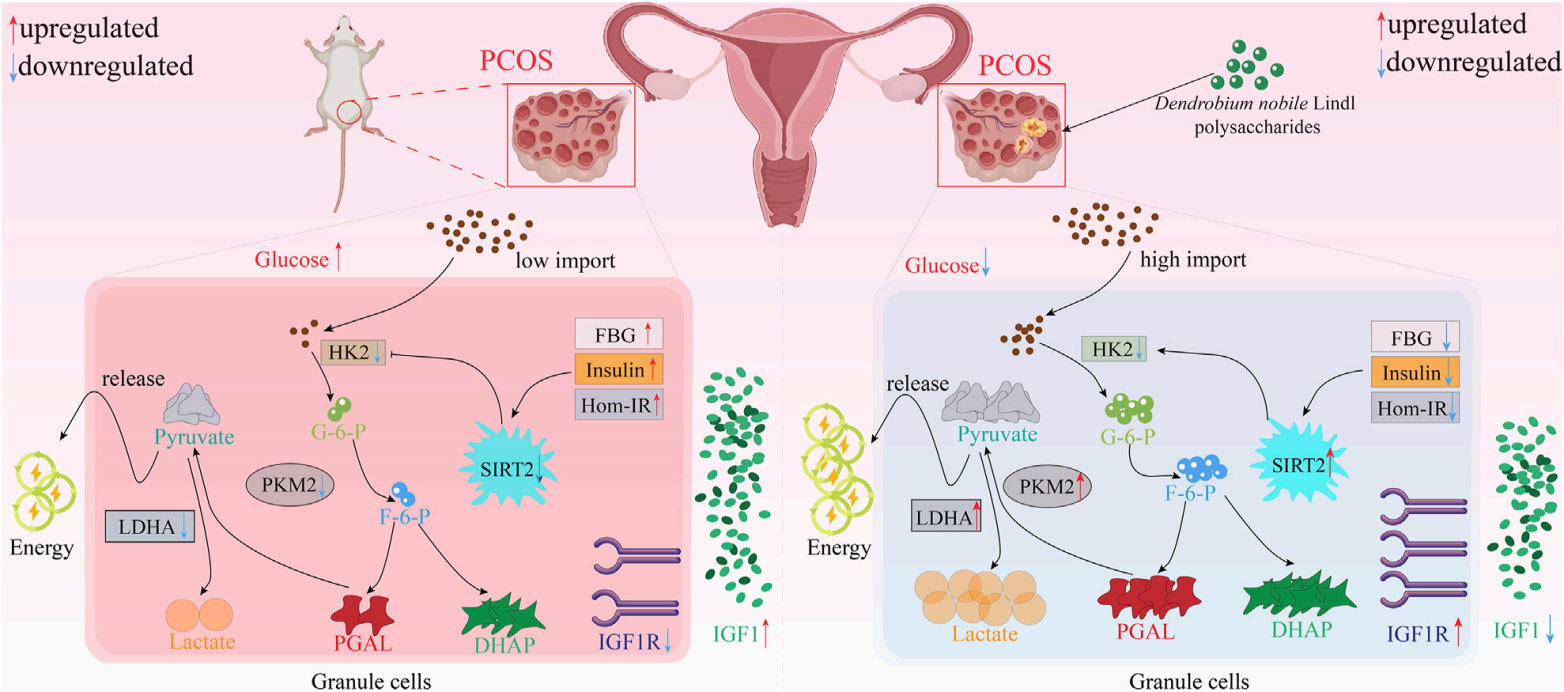
Potential mechanism of polysaccharides from *D. nobile* in ovarian protective effect.

Insulin resistance is common in PCOS. *D. nobile* polysaccharides (DNP) can regulate IR in granulosa cells of PCOS. In the situation when DNP (200 mg/kg/day per day for 30 days) was administered to PCOS rats, the body weight and ovarian weight were reduced, normal estrous cycle was restored, and hormonal levels and ovarian morphology were returned to normal. Moreover, DNP significantly decreased the level of serum glucose, insulin, HOMA-IR, and restored the expression of IGF1 and IGF1R in PCOS rats. These results were substantiated histologically by immunohistochemistry (IHC). Comprehensively, these results suggest that DNP intervention has the potential to rectify IR in rats with PCOS. Also, DNP intervention can also promote granulosa cells (GCs) glycolysis and improve the mechanism of follicular dysplasia. The effect of DNP on insulin sensitivity and glycolysis process of KGN cells treated with insulin further confirms this result. Interestingly, SIRT2 might be the key for DNP to regulate the glycolysis rate of granulosa cells (Hu et al., 2024; Liang et al., 2023; Martin and Madassery, 2006) (Figure 6).

### 4.4 Gastric protective effect

Drinking alcohol is a prevalent social activity, but it can also cause many diseases (Patel et al., 2022). Alcohol-mediated injury is particularly prone to occur in gastrointestinal tract. Alcohol passes out rapidly along the esophagus, into the stomach and the small intestine and finally into the bloodstream through capillaries. This conduct has a direct impact on the epithelial cells of the gastric mucosal, breaking the protective mucosal barrier and causing acute gastric congestion, erosion, and, in some cases, superficial ulceration (Sun et al., 2025). Zhang et al. (2018) assessed the therapeutic effect and mechanism of the *D. nobile* polysaccharides (JCP) on ethanol-induced gastric injury in rats. The forty male Wistar rats were provided randomly into five groups: the control group, vehicle group, omeprazole group, low-dose (L-JCP, 100 mg/kg) and high-dose (H-JCP, 300 mg/kg) JCP groups. After 28 days of continuous gavage administration, all groups except the control group were induced with gastric ulcers through gavage with 100% ethanol (4 mL/kg). Gastric ulcer evaluation followed by histological analysis revealed that the ulcer indices for omeprazole, L-JCP, and H-JCP pretreated rats were 0.17% ± 0.16%, 0.78% ± 0.26%, and 0.32% ± 0.19%, respectively (*P* < 0.05). Compared to the vehicle group, JCP pretreatment reduced gastric hemorrhage and the occurrence of inflammatory cells. Intragastric administration of ethanol decreased the expression of SOD and increased MDA content in normal rats, whereas the H-JCP group showed a significantly higher SOD expression and low MDA accumulation. EGF, one of the major cell-protective growth factors that promote mucosal blood flow and epithelial cell proliferation, was considerably upregulated in the rats that were given 300 mg/kg JCP. Western blot analysis indicated that H-JCP group inhibited the expression of p-JNK, p-ERK, MMP-2 and MMP-9 in gastric tissue (Zhang et al., 2018; Zhang et al., 2020).

### 4.5 Neuroprotective effect

Vascular dementia (VD) is a cerebrovascular disease characterized by cognitive impairment and is an also common form of dementia. Its incidence rate has continued to rise in recent years. Its pathophysiology involves a series of events triggered by blood-brain barrier (BBB) disruption, endothelial dysfunction, and microcirculation failure, leading to neuronal energy depletion and excitotoxicity (Wang Y. et al., 2025). However, currently there are limited treatment options for this disease, which is a major challenge for the medical community (Zhong et al., 2024). Ferroptosis, a form of cell death driven by iron, is characterized by iron overload and excessive lipid peroxidation. Iron deposition and lipid peroxidation are key pathological features in neurodegenerative diseases (Ali et al., 2025). The explanation of ferroptosis has created new prospects in the study of the pathophysiology and therapeutic potential of VD (Ji et al., 2022). *D. nobile* polysaccharides (DNP) have the capacity to mitigate ferroptosis and maintain cognitive ability in VD rats. The current research involved 72 males of SD rats that were randomly divided into a sham operation group, a VD group, and a DNP group (100 mg/kg/day). The model and DNP groups were subjected to permanent ligation of the bilateral common carotid arteries, and an equal procedure was done on the sham operation group without the carotid artery ligation. The Morris water maze (MWM) test was used to examine the escape latency and the number of platform crossings. The tissue of the hippocampus was dissected after the end of the MWM experiment to determine GSH, GPx4, xCT, and PSD-95 expression and and mitochondrial morphology and ultrastructure were observed using transmission electron microscopy (TEM). The DNP group had significantly shorter escape latency (*P* < 0.05) and increased performance of platform crossing (*P* < 0.05) compared with the model group. The TEM analysis demonstrated that there are increased structures of the mitochondrial membranes and cristae in the DNP group. The GSH, xCT, and GPx4 levels in the hippocampus were increased in this group (*P* < 0.01). Besides, there was an increase of the synaptic vesicles, synaptic active zone (SAZ) length, thicker postsynaptic densities (PSD) and PSD-95 protein expression in DNP-treated rats. These results suggest that DNP can improve cognitive function in VD by alleviating ferroptosis (Hossain, 2009; Ming et al., 2023).

It is worth mentioning that DNP can also improve behavioral disorders caused by spinal cord injury (SCI) by inhibiting ferroptosis. The Basso-Beattie-Bresnahan scores of motor function in the DNP group were lower than those in the sham operation group (*P* < 0.05) and higher than those in the model group (*P* < 0.05) after 7, 14, 21, and 28 days of treatment. TEM observed that the mitochondrial membrane of the model group was damaged, and the mitochondrial cristae almost disappeared. However, after DNP treatment, there was improvement and a decrease in iron content (24 and 48 h after injury). The HE stains after 28 days of treatment showed that the spinal cord tissue defect area in the DNP group was smaller compared to the model group. And compared with the sham operation group, the xCT, GSH, Gpx4, and GRSFI in the spinal cord tissue of the model group rats decreased (*P* < 0.05), while the expression of these indicators increased after DNP treatment. NeuN^+^ is a commonly used marker for mature neurons. DNP treatment increased NeuN^+^ cells at 14 and 28 days after SCI (Huang J. et al., 2024; Wei et al., 2024). DNP has neuroprotective effects and can improve some neurological diseases by alleviating or inhibiting ferroptosis.

### 4.6 Anti-inflammatory effect

Inflammation is an automatic defense response of the body. It is closely related to the occurrence and development of many diseases, including arthritis, inflammatory bowel disease, diabetes, nervous system diseases, etc (Han et al., 2024; Lee et al., 2024). Therefore, studying the anti-inflammatory effects of polysaccharides from *D. nobile* can provide reference and inspiration for the treatment of these diseases. The important regulatory cells in the inflammatory process are macrophages. Chen et al. (2022) investigated the anti-inflammatory effects of *D. nobile* polysaccharides (DNP1 and DNP2) using lipopolysaccharide (LPS) - induced RAW264.7 macrophages as a model. Firstly, treatment of RAW264.7 macrophages with DNP1 and DNP2 at concentrations of 12.5, 25, 50, 100, and 200 μg/mL showed no cytotoxicity and significant cell proliferation. LPS stimulation can excessively increase the levels of inflammatory factors such as NO, TNF-α, IL-1β, IL-6 in RAW264.7 macrophages, inducing inflammatory responses, while DNP1 and DNP2 significantly reduce the production of inflammatory factors in a dose-dependent manner (Chen et al., 2022; Pan et al., 2014). The study is used to provide a theoretical basis of producing and using new products containing DNP1 and DNP2 with the aim of relieving inflammation. The team then investigated the mechanism through which DNP1 exerts anti-inflammatory effects, using surface plasmon resonance (SPR) binding assay, molecular docking techniques, and macrophage receptor blockade experiments. From the binding kinetic parameters and sensor graph of SPR, the dissociation constant value of DNP1 is 0. Molecular-docking technology has revealed that the binding energy of the DNP1 and TLR4-MD2 is −7.9 kcal/mol. The results of macrophage receptor blockade experiments showed that after treatment with different concentrations of DNP1, there were no significant changes in the release of NO, TNF-α, IL-1β, IL-6 from RAW 264.7. It is speculated that DNP1 may exert immunomodulatory effects by binding to the TLR4-MD2 complex and inhibiting the TLR4-MD2 mediated signaling pathway (Li et al., 2024; Zhang et al., 2023).

### 4.7 Anti-viral effect

Plant viruses parasitize in plants and can affect their normal growth and development (Yuan et al., 2022). For crops, it also causes a decrease in their quality and yield. To explore more antiviral agents, researchers measured the anti-cucumber mosaic virus (CMV) and anti-tobacco mosaic virus (TMV) effects of 125 μg/mL^−1^ DNPE6 (4) in tobacco using chitosan oligosaccharides (COS) and lentinan as controls. The protective activities of DNPE6 (4) against CMV and TMV were 40.4% and 69.9%, respectively. Among them, DNPE6 (4) has better protective activity against TMV than COS (39.1%) and lentinan (52.3%). After treatment with DNPE6 (4), NADPH oxidase and NAD(P)H increased. In addition, DNPE6 (4) can enhance the activity of defensive enzymes (POD, PAL, SOD) in tobacco. The PALS enzyme activity of the CK + DNPE6 (4) group reached its peak on the first day, while SOD and POD enzyme activities reached their peak on the third day. Moreover, the enzyme activities that reached their peak in the CK + DNPE6 (4) group were higher than those of the CK group. The enzyme activities of PALS and POD in the TMV + DNPE6 (4) treatment group peaked on third day, while that of SOD enzyme peaked on fifth day. A similar tendency was noted in the current study: the activity of enzymes was the highest in the TMV + DNPE6 (4) group and were higher than those in the TMV group. The above implies that DNPE6 (4) can effectively induce the host to produce defensive enzymes to resist plant virus infection. The effect of DNPE6 (4) on the expression of defense related genes was detected by RT-PCR. The results showed that DNPE6 (4) could increase the expression level of SOD and the expression levels of ICSI, EDSI, and PRI up-stream and down-stream of the Salicylic acid (SA) pathway, while inhibiting the protein expression of CAT. This information shows that DNPE6 (4) can accumulate SA and induce systemic acquired resistance (SAR). In summary, DNPE6 (4) exhibits anti-CMV and anti-TMV effect (Li et al., 2019). Subsequently, the antiviral effect of DNPE6 (11) against CMV, TMV, and potato virus Y (PVY) were studied. DNPE6 (11) has significant therapeutic and inactivation activity against CMV, significant protective effect against PVY, and greater efficacy than that of the control Ningnanmycin. In addition, preliminary mechanistic studies have found that DNPE6 (11) has superior binding ability with cucumber mosaic virus coat protein (Li et al., 2020; Mohan et al., 2020). Taken together, DNPE6 (4) and DNPE6 (11) can be deemed as potential antiviral compounds.

## 5 Structure-activity relationships of *D. nobile* polysaccharides

Understanding the relationship between structure and activity not only deepens insights into the biological roles of polysaccharides but also provides a scientific foundation for their applications and offers valuable guidance for the study of other compounds (Yao et al., 2024; Zhang Y. et al., 2024). *D. nobile* polysaccharides consist of various monosaccharides, and the differences in their monosaccharide composition result in diverse pharmacological effects (Table 4). For instance, DNP1, which contains Man and Glc, exhibits anti-inflammatory effect (Pan et al., 2014). In contrast, DNP, consisting of Man, Glc, and Gal, shows protective effects on testicular tissue in DM (Fan et al., 2023; Luo et al., 2022). Even with the same monosaccharide composition, its pharmacological effects may vary due to differences in monosaccharide ratios. For example, DNLP and DNP both have Man: Glc: Gal in ratios of 26.9%: 66.2%: 6.9% and 12.49%: 65.2%: 6.4%, respectively. DNLP has a protective effect on polycystic ovary syndrome (Song et al., 2022; Zhang et al., 2020). DNP can regulate insulin resistance in granulosa cells of polycystic ovary syndrome (Hu et al., 2024; Martin and Madassery, 2006). Despite having the same monosaccharide components (Rha, Ara, Xyl, Man, Glc, Gal), DNLP exhibits anti-photoaging effects, whereas DNP acts as an anti-oxidant (Li et al., 2022; Luo et al., 2009). Moreover, the pharmacological effects of *D. nobile* polysaccharides depend on their Mw. For example, the Mw of JCS1 is 23 kDa, while that of its acetylated derivative YJCS1 is 18.8 kDa. Studies comparing the biological activity of these two polysaccharides in PC-12 cells show that YJCS1 induces neuron production, while JCS1 does not (Jin et al., 2017).

**TABLE 4:** The structural characteristics and corresponding biological activities of *D. nobile* polysaccharides.

Polysaccharide name	Mw (kDa)	Ara (%)	Fuc (%)	Gal (%)	GalA (%)	Glc (%)	GclA (%)	Man (%)	Rha (%)	Xyl (%)	Structural related information	Biological activity	Ref
DNLP	N/A	17.76	0	12.38	0	19.70	0	11.63	10.76	27.77	N/A	Anti-photoaging effect	Li et al. (2022)
DNLP	1.2–11.2	0	0	6.9	0	66.2	0	26.9	0	0	N/A	Ovarian protective effect	Zhang et al. (2020)
DNP	13.2	0	0	6.4	0	65.2	0	12.49	0	0	N/A	Ovarian protective effect	Hu et al. (2024)
DNP	106.6	0	0	3.5	0	68.5	0	23.2	0	0	N/A	Testicular protective effect	Luo et al. (2022)
DNP1	67.72	0	0	0	0	24.14	0	75.86	0	0	Straight-chain glucomannan *β*-(1 → 4)	Anti-inflammatory effect	Li et al. (2024), Chen et al. (2022)
DNP2	37.45	0	0	0	0	27.68	0	72.32	0	0	N/A	Anti-inflammatory effect	Chen et al. (2022)
DNPE6 (4)	99.2	55.56	0	6.67	0	20	0	17.78	0	0	Neutral homogeneous	Anti-viral effect	Li et al. (2019)
DNPE6 (11)	3.01	0	0	17.65	0	64.71	0	17.65	0	0	Acetylated polysaccharide	Anti-viral effect	Li et al. (2020)
DNP-W4	500	0	0	23.15	8.33	45.37	0	9.26	9.26	4.63	Acidic polysaccharide *β*-(1 → 4), *β*-(1 → 6)	Immunomodulatory effect	Wang et al. (2017)
JCS1	23	2.25	0	0	0	90.34	0	4.49	0	2.92	Mannoglucan *α*-(1 → 4), *β*-(1 → 4)	N/A	Jin et al. (2017)
JCS1S2	N/A										Sulfated polysaccharide *α*-(1 → 4), *β*-(1 → 4)	Anti-angiogenesis effect	Wang et al. (2019)
YJCS1	18.8	N/A									Acetylated polysaccharide	Neuritogenesis induced effect on PC-12 Cells	Jin et al. (2017)
DNP	87.6	1.50	0	17.05	0	63.22	0	16.51	0.54	1.18	*α*-(1 → 4), *α*-(1 → 6)	Antioxidant effect	Luo et al. (2009)
*D. nobile* polysaccharide	85.72	1.05	0	1.6	0	48.63	0	48.73	0	0	N/A	Antioxidant effect	Liu et al. (2019)

N/A, not available.

Polysaccharides with either excessively high or low Mw may fail to exert their biological activity effectively. High Mw polysaccharides struggle to enter cells via the cell membrane, while low Mw polysaccharides, despite being able to bind to active sites, may lack the complex spatial structures necessary for effective biological function (Wang et al., 2023). As Mw is a structural feature that can be regulated, further research on *D. nobile* polysaccharides with varying Mw will help identify the optimal Mw range for robust biological activity, contributing significantly to understanding the structure-activity relationship. Interestingly, *D. nobile* polysaccharides can exhibit similar pharmacological effects despite differences in Mw and monosaccharide composition. For example, both DNPE6 (4) and DNPE6 (11) demonstrate anti-viral activity, even though their Mw differ substantially, at 99.2 and 3.01 kDa, respectively. DNPE6 (4) is composed of Ara, Glc, Gal, and Man, while DNPE6 (11) consists of Man, Glc, and Gal. These similar pharmacological effects may be attributed to the presence of shared structural motifs, such as →4)-d-Glc*p*-(1→ and →1)-d-Man*p*-(3,6→) (Li et al., 2019; Li et al., 2020).

Modifying polysaccharides is a key strategy for enhancing their development and utilization. Fan et al. (2020) applied non-thermal plasma treatment to JCS1. The results demonstrated a significant increase in the hydrophilicity of the polysaccharides and a notable improvement in their phagocytic ability towards RAW264.7 cells. Additionally, the treatment stimulated the secretion of cytokines, including TNF-α, IL-6, and IL-1, thereby enhancing their immune activity *in vitro*. Furthermore, studies have indicated that the JCS1 does not promote the extension of neural processes in PC-12 cells. However, its acetylated derivative, YJCS1, was found to induce neuronal differentiation in these cells. The sulfated polysaccharide JCS1S2 has shown effects on angiogenesis by modulating the VEGF pathway. Specifically, it inhibits the expression of VEGF and its transcription factor AP-1, thereby suppressing angiogenesis (Fan et al., 2020; Jin et al., 2017; Wang et al., 2019).

Currently, research on *D. nobile* polysaccharides varies in focus and depth. Due to the complex and diverse structural characteristics of these polysaccharides, and the variability in extraction methods across different studies, challenges remain in establishing clear structure-activity relationships. These challenges make it difficult to carry out comprehensive and reliable analyses. To better understand the structure-activity relationship of *D. nobile* polysaccharides, it is recommended that an exchange platform for research data be established. This platform would integrate data from various research teams, enabling comprehensive analysis and improving research efficiency and result reliability. Such an initiative would also help bridge gaps and address differences in knowledge, methodologies, and experimental conditions across different research groups. In conclusion, while research on the structure-activity relationship of *D. nobile* polysaccharides is still in its infancy, further innovation in high-purity polysaccharide preparation techniques, as well as strengthened foundational research into their mechanisms of action, is essential to advancing polysaccharide research.

This platform would integrate data from numerous research groups, and this would facilitate careful analysis and increase the efficiency of research and accuracy of results. This endeavor would also further narrow differences and harmonize differences in knowledge, approaches, and conditions of experiments within and across different research groups. In all, while doing research on the structure-activity relationship of *D. nobile* polysaccharides is still in its infancy, further breakthrough in high-purity preparation methods of polysaccharides, and more thorough foundational research into their mechanisms of action is needed to further advance polysaccharide science.

## 6 Technological prospection of *D. nobile* polysaccharide

**FIGURE 7:**
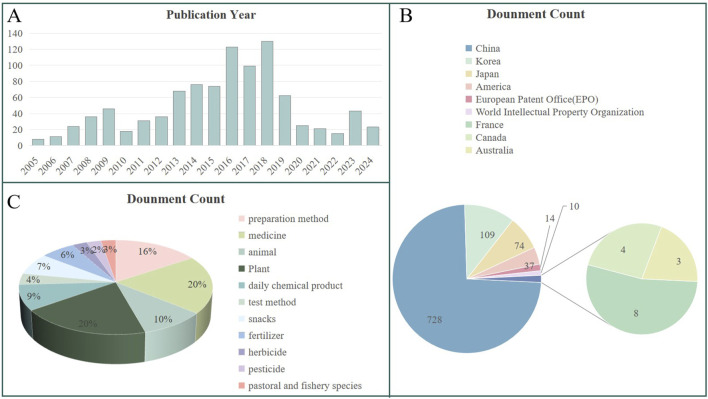
Analysis of *D. nobile* polysaccharides patents searched in https://t.incopat.com: **(A)** Number of patent applications per year. **(B)** Geographical ranking of patents. **(C)** Technical usage distribution.

Search for patent applications related to *D. nobile* polysaccharides using the search terms “*Dendrobium nobile* Lindl.,” “Polysaccharides,” and “*Dendrobium nobile* Lindl. Polysaccharides.” Data reveals an upward trend in patent filings from 2010 to 2017, followed by a decline, with a slight fluctuation around 2020. Key countries involved in these applications include China, Japan, Korea, and Canada, with China accounting for approximately 74% of the applications, followed by Korea and Japan. Other countries or regions contribute to a smaller extent. As depicted in Figure 7C, the primary technical applications of *D. nobile* polysaccharides are classified under “medicine” and “preparation method”. The substantial proportion of patents related to “medicine” reflects the growing recognition of the medicinal value of *D. nobile* polysaccharides, which are increasingly being researched and developed. In addition, patents related to “preparation methods” highlight ongoing challenges such as low extraction rates, low purity, and high production costs. Optimizing these processes is crucial to improving the competitiveness of *D. nobile* polysaccharides products. Through technological advancements and process optimization, the market position of these products can be strengthened.

To our knowledge, the commercialization of polysaccharides related to *D. nobile* is still in the exploratory stage. However, some application technology patents related to *D. nobile* polysaccharides have been made public. A patent (CN113413329A) shows that *D. nobile* polysaccharides can be used for making face cream. *D. nobile* polysaccharides have the effect of anti-oxidation and promoting the growth of fibroblasts. It is added with some other raw materials (such as Vaseline, glycerin monostearate, propylene glycol) in proportion to make face cream. Long term external applications can protect the skin and help remove harmful substances from the skin. In addition, a patent (CN115607564A) shows that polysaccharides from *D. nobile* can also be used in the preparation of drugs or daily chemical products for preventing and treating skin photoaging. Besides, *D. nobile* polysaccharides can also be used in the food industry. Patent CN110343191A shows that *D. nobile* polysaccharides can be used to make solid beverages. This beverage not only has the flavor and nutrition of *D. nobile*, but also has the effect of enhancing immunity, which meets the consumer demand under the influence of healthy diet.

## 7 Summary and prospect

*D. nobile*, a traditional Chinese medicine, is classified as a second-class protected plant in China due to its rarity, harvesting difficulty, and low yield. This status highlights the need for comprehensive research to fully exploit its potential value. The efficacy of traditional Chinese medicine is closely associated with its phytochemical composition (Yu et al., 2024). In the field of plant chemistry, both small molecule compounds and large molecule compounds are research objects. After investigation, it was found that most research has focused on small molecule compounds of *D. nobile*, until recent years when researchers began to be interested in polysaccharides (Fan et al., 2023). The development of extraction technology has led to variety of ways that are used to isolate these polysaccharides, and various processes produce polysaccharides with different structural features and biological activity. *D. nobile* polysaccharide has made notable progress in the research of its structure and biological activity, especially in the aspects of anti-photoaging, improvement of complications of diabetes mellitus, and ovarian protective effect. However, research on *D. nobile* polysaccharides still faces numerous challenges.

Isolation and purification of *D. nobile* polysaccharides is now a tedious and lengthy process. Modern studies focus on increasing the efficiency of extraction and purity of polysaccharide through the optimization of the existing protocol, or also by implementing additional methods (Chen M. et al., 2024; Ledesma-Escobar et al., 2015). Even though some of the emerging extraction technologies have been proposed in the recent past, their majority are still at the level of development, and the quality of *D. nobile* polysaccharides cannot be guaranteed without uniform protocols. Improving the existing separation techniques and legislatively unifying these procedures will therefore become a necessity in the following stage of *D. nobile* polysaccharide research. The pharmacological activity of *D. nobile* polysaccharides has been supported by cell and animal experimental data, but in order to comprehensively evaluate their biological processes and generate strong scientific data, deeper experiments need to be designed, including human experiments when necessary. The anti-photoaging potential of *D. nobile* polysaccharides has been already pointed out through experiments, making them potential natural resources with regard to skin photoaging prevention and treatment. This empirical background is the basis of creating new skincare products or pharmaceuticals. In addition, there is limited research on the absorption, distribution, and metabolism of *D. nobile* polysaccharides within the body. However, advancements in detection technologies such as immunoassays, fluorescence, and isotope labeling are making the *in vivo* pharmacokinetics of polysaccharides more apparent. Some studies indicate that polysaccharides exert beneficial pharmacological effects when administered orally. Conducting systematic studies on the absorption and metabolic pathways of *D. nobile* polysaccharides, coupled with comprehensive investigations, will be instrumental in promoting its industrialization. With technological advancements, *D. nobile* polysaccharides will present broader applications, bringing profound societal benefits.

## Abbreviations

Ara, Arabinose; DM, Diabetes mellitus; Fuc, Fucose; Gal, Galactose; GPC, Gel permeation chromography; Glc, Glucose; HPGPC, High-performance gel permeation chromography; HWE, Hot water extraction; MWM, Morris water maze; NMR, Nuclear Magnetic Resonance; PEG, Polyethylene glycol; PCOS, Polycystic ovary syndrome; ROS, Reactive oxygen species; RSM, Response surface methodology; SWE, Subcritical water extraction; UAE, Ultrasound assisted extraction; VD, Vascular dementia; UV, Ultraviolet.
